# Prognostic significance of an innovative staging system based on the logarithmic odds of positive lymph nodes for resectable gastroesophageal cancer after neoadjuvant chemoradiation: a population-based study with external validation of data

**DOI:** 10.1186/s12967-024-05448-5

**Published:** 2024-08-29

**Authors:** Shuang Liu, Zhengmiao Wang, Yanyan Ge, Yixuan Zhao

**Affiliations:** Department of Ultrasound, The Second Hospital of Jilin University, Changchun, 130000 China

**Keywords:** Neoadjuvant chemoradiation, Gastroesophageal cancer, LODDS, Staging, OS

## Abstract

**Background:**

After receiving neoadjuvant chemoradiation, the number of examined lymph nodes in resectable gastroesophageal cancer (GEC) will decrease, this may not accurately determine the N staging. So our study evaluates the clinical significance of a new staging model based on the logarithmic odds of positive lymph nodes (LODDS) in patients with GEC after receiving neoadjuvant chemoradiation.

**Methods:**

A total of 1 130 patients with pathologically diagnosed GEC who received neoadjuvant chemoradiation from 2004 to 2019 included in the National Cancer Institute Surveillance, Epidemiology, and Results (SEER) database were selected for analysis. Lymph nodes were staged according to the AJCC TNM staging system (eighth edition) and LODDS. Patient prognosis across the two systems were evaluated by the Kaplan–Meier method, differences in node staging were evaluated by the Akaike information criterion and Bayesian information criterion. In addition, 914 patients from our center were externally validated.

**Results:**

Compared to the traditional TNM staging system, the new TLODDSM staging system was comprised of stage I, stage II, stage IIIA, stage IIIB, and stage IVA, and decision curve analysis showed that the new staging system had higher benefits for different decision thresholds than the old staging system. The Akaike information criterion and Bayesian information criterion of the new staging system was lower than those of the old staging system, indicating the sensitivity of the TLODDSM staging system for predicting the prognosis of patients was higher. In addition, stage-IIIB or -IVA patients in the new staging system benefited from adjuvant chemotherapy. The externally validated data from our center supported this conclusion.

**Conclusions:**

Compared to the TNM staging system, the TLODDSM staging system has significant advantages in predicting prognosis of patients with GEC who have completed neoadjuvant chemoradiation, guiding the adjuvant chemotherapy for patients.

## Background

The latest global cancer statistics reveal that gastroesophageal cancer (GEC) is the ninth most common malignancy and the sixth most common cause of cancer-related death [1]. Primary esophageal and GEC can be classified as either adenocarcinoma or squamous cell carcinoma. Caucasians in western countries mainly have adenocarcinoma, whereas Caucasians in eastern countries mostly have squamous cell carcinoma. Presently, GEC is treated by surgery, and although significant progress has been made in the treatment of GEC, the overall 5-year survival rate of patients remains low, which may be due to the lack of an accurate staging system before treatment and a standard treatment protocol [2, 3].

Different stages of GEC require different treatments. Patients with early stages of the disease can be treated with surgery, those with locally advanced stages can be treated with preoperative radiotherapy and chemotherapy, and those with distant metastases can receive palliative care to improve symptoms and quality of life. The prognosis of patients with GEC at different stages varies greatly [4, 5]. Cunningham et al. reported that among patients with resectable GEC, those receiving neoadjuvant chemotherapy had better overall survival (OS) (hazard ratio [HR] = 0.75; 95% CI 0.60–0.93; P < 0.01) and progression-free survival (PFS) (HR = 0.66; 95% CI 0.53–0.81; P < 0.01) compared to those treated with surgery alone [6]. As neoadjuvant chemoradiation replaces EGC resection, the pathological staging of advanced cancer is losing its clinical relevance, although it can still be used as a reference for staging early disease and predicting patient survival. Moreover, neoadjuvant chemoradiation associates with a decreased number of examined lymph nodes (ELNs). The eighth edition of NCCN includes TNM staging for GEC patients who received neoadjuvant chemoradiation; however, there are disadvantages to using this staging system because of the decreased number of ELNs [7]. In addition, it is unclear whether this population can benefit from adjuvant chemotherapy (ACT). Therefore, we propose a new TNM staging system based on the logarithmic odds of positive lymph nodes (LODDS), which provides more accurate pathological staging of patients with GEC who have received neoadjuvant treatment and selects the population who can truly benefit from ACT. The currently recognized comprehensive treatment for GEC still recommends ACT as the recommended treatment method. Nano-activated carbon can play the role of lymph node tracer in the operation of gastric cancer, thyroid cancer, pancreatic cancer and other malignant tumors. Nano-activated carbon also has the same adsorption characteristics as carbon, has a larger specific surface area. Therefore, it can be used as a carrier to adsorb chemotherapy drugs for lymphatic system targeted chemotherapy. Green nanomaterials are the direction of future treatment [8–10].

This study included data from the SEER database of patients with resectable GEC who received neoadjuvant chemoradiation. The patients were divided into five groups according to the LODDS in order to evaluate the accuracy and relevance of the new staging system compared to the old staging system in predicting the prognosis of patients. The benefits of the new staging system were validated through external data, and the population that could benefit from ACT was confirmed.

## Methods

### Patient cohort

SEER*Stat (version 8.4.0) software was used to identify 1 444 patients with resectable GEC confirmed between 2004 and 2019. The inclusion criteria were as follows: (1) patients with pathologically confirmed GEC (ICD-O-3: C16.0), (2) patients with complete follow-up and survival data, (3) patients who received neoadjuvant chemotherapy or radiotherapy, (4) patients who underwent radical surgery, and (5) patients with resectable primary GEC. The variables were age, gender, race, grade, tumor size, tumor stage, ELN, positive examined lymph nodes (PLN), and survival information. Patients were excluded if data were missing. The externally validated data consisted of 914 patients with resectable GEC who received neoadjuvant chemoradiation and underwent surgery in our department from 2011 to 2018. The staging of all tumors was according to the TNM staging system (eighth edition).

### Statistical analysis

The distribution of pathological factors was examined, and the TNM staging system was replaced with the LODDS staging system, which was defined as log [(PLN + 0.5)/(ELN-PLN + 0.5)]. LODDS values ranged from − 2.1 to 1.8, with an inter-group spacing of 0.2. Patients were divided into 17 groups, and the HR (range, − 2.1 ≤ LODDS ≤ − 2.0) was defined as 1 and sorted from low to high. The OS of the adjacent two groups was subjected to log rank test, and 4 chi square values with significant differences were selected from 16 chi square values. The 17 groups were divided into five stages. LODDS1 (− 2.1–1.8) consisted of 56 individuals, LODDS2 (− 1.7–1.4) consisted of 395 individuals, LODDS3 (− 1.3–0.2) consisted of 505 individuals, LODDS4 (− 0.1–0.2) consisted of 82 individuals, and LODDS5 (0.3–1.8) consisted of 92 individuals. The survival curves were compared among the five groups, and an improved TLODDSM staging system (I-IVA) was established by replacing the N staging system in the AJCC staging system (eight edition).

When evaluating the discriminative ability of the two staging systems for prognosis, we used the Akaike information criterion (AIC) and Bayesian information criterion (BIC). We used decision curve analysis (DCA) to evaluate the net clinical benefit of the new staging system and compared it with the AJCC staging system (eight edition). We used chi-square test to evaluate the prognostic homogeneity of the two staging systems (the larger the likelihood ratio, the better the prognostic homogeneity of the staging system) and survival curve and COX analysis to analyze the prognosis of patients at different stages. All statistical analyses were conducted using SPSS (version 24.0) and R language (version 4.0.0) software packages with P < 0.05 indicating statistically significant differences.

## Results

### Patient demographics

**Fig. 1:**
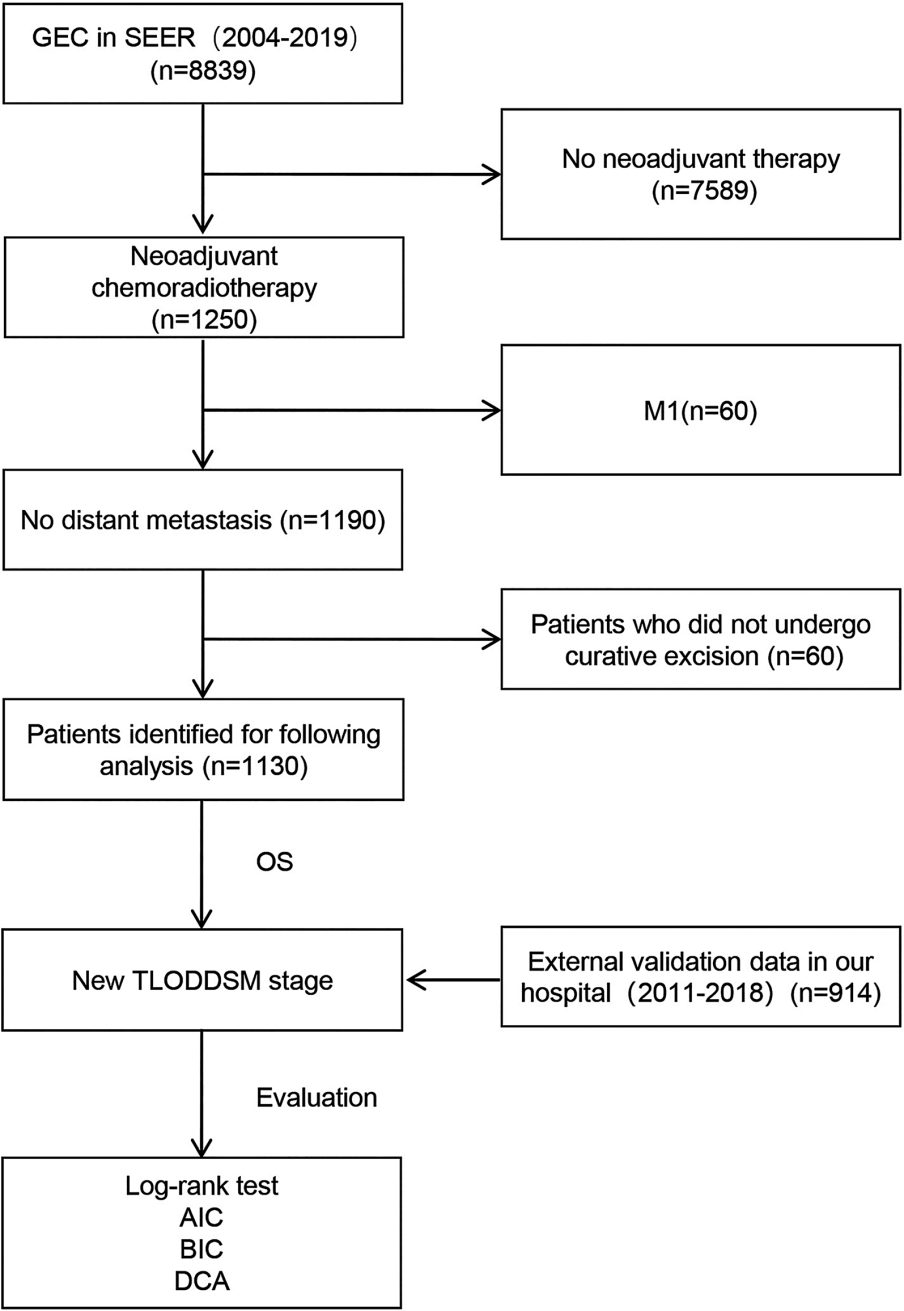
Flowchart of the selection process of included patients

A total of 1130 patients with resectable GEC who received neoadjuvant chemoradiation were included (Fig. 1). Specifically, 516 (45.7%) patients were 65 years of age or older, 168 (14.9%) patients were female, 290 (25.7%) patients had poorly differentiated tumors, 410 (36.3%) patients had tumors greater than 5 cm in diameter, and 88 (7.8%) patients had T4 tumors. The median ELN was 14, whereas the median PLN was 1 (Table 1). The median survival period was 27 months (range, 0–190 months), and the number of deaths was 590 (52.2%).

**Table 1:** Characteristics of patients

Variable	Training [n (%)]	External [n (%)]	P value
Age			< 0.001
< 65	614 (54.3)	617 (67.5)	
≥ 65	516 (45.7)	297 (32.5)	
Sex			< 0.001
Male	962 (85.1)	646 (70.7)	
Female	168 (14.9)	268 (29.3)	
Race			< 0.001
White	1009 (89.3)	0 (0.0)	
Black	21 (1.9)	0 (0.0)	
API	75 (6.6)	0 (0.0)	
Other	25 (2.2)	914 (100.0)	
Grade			< 0.001
Well	348 (30.8)	203 (22.2)	
Moderately	492 (43.5)	298 (32.6)	
Poorly	290 (25.7)	413 (45.2)	
Size (cm)			< 0.001
≤ 5	642 (56.8)	537 (58.8)	
> 5	410 (36.3)	377 (41.2)	
Unknown	78 (6.9)	0 (0.0)	
T stage			< 0.001
T0	284 (25.1)	10 (1.1)	
Tis	54 (4.8)	7 (0.8)	
T1	434 (38.4)	158 (17.3)	
T2	174 (15.4)	132 (14.4)	
T3	96(8.5)	135 (14.8)	
T4a	71 (6.3)	458 (50.1)	
T4b	17 (1.5)	14 (1.5)	
ELN count, median	14	18	> 0.05
Positive ELN count, median	1	2	> 0.05

*API* Asian/Pacific Islander, *ELN* examined lymph nodes

### New lymph node staging system

**Fig. 2:**
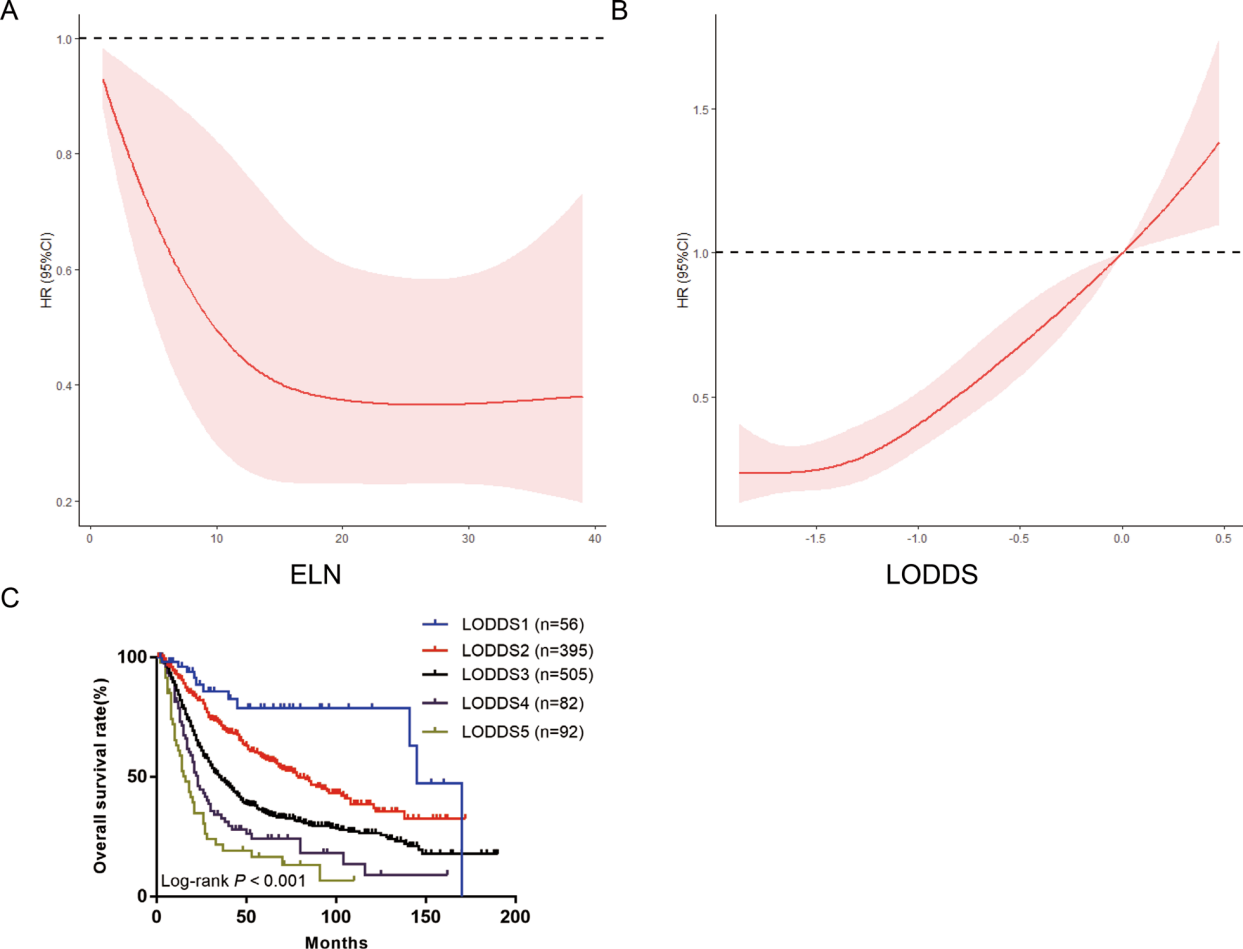
**A** hazard estimates of death from ELN; **B** hazard estimates of death from LODDS; **C** The Kaplan–Meier curves of OS for patients in our new LODDS staging system

**Fig. 3:**
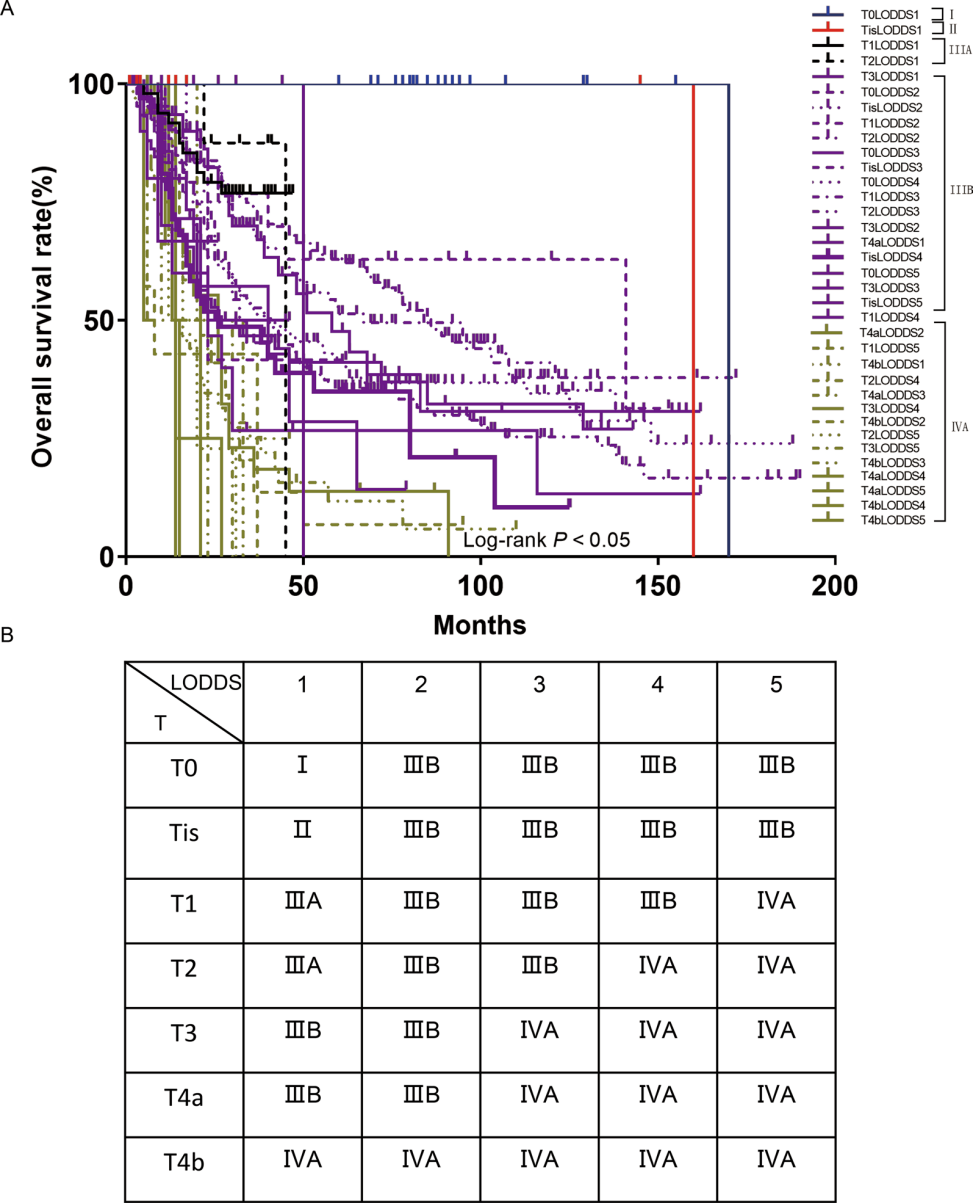
**A** The Kaplan–Meier curves of OS for patients in our new LODDS staging system; **B** The new TLODDSM staging system

**Fig. 4:**
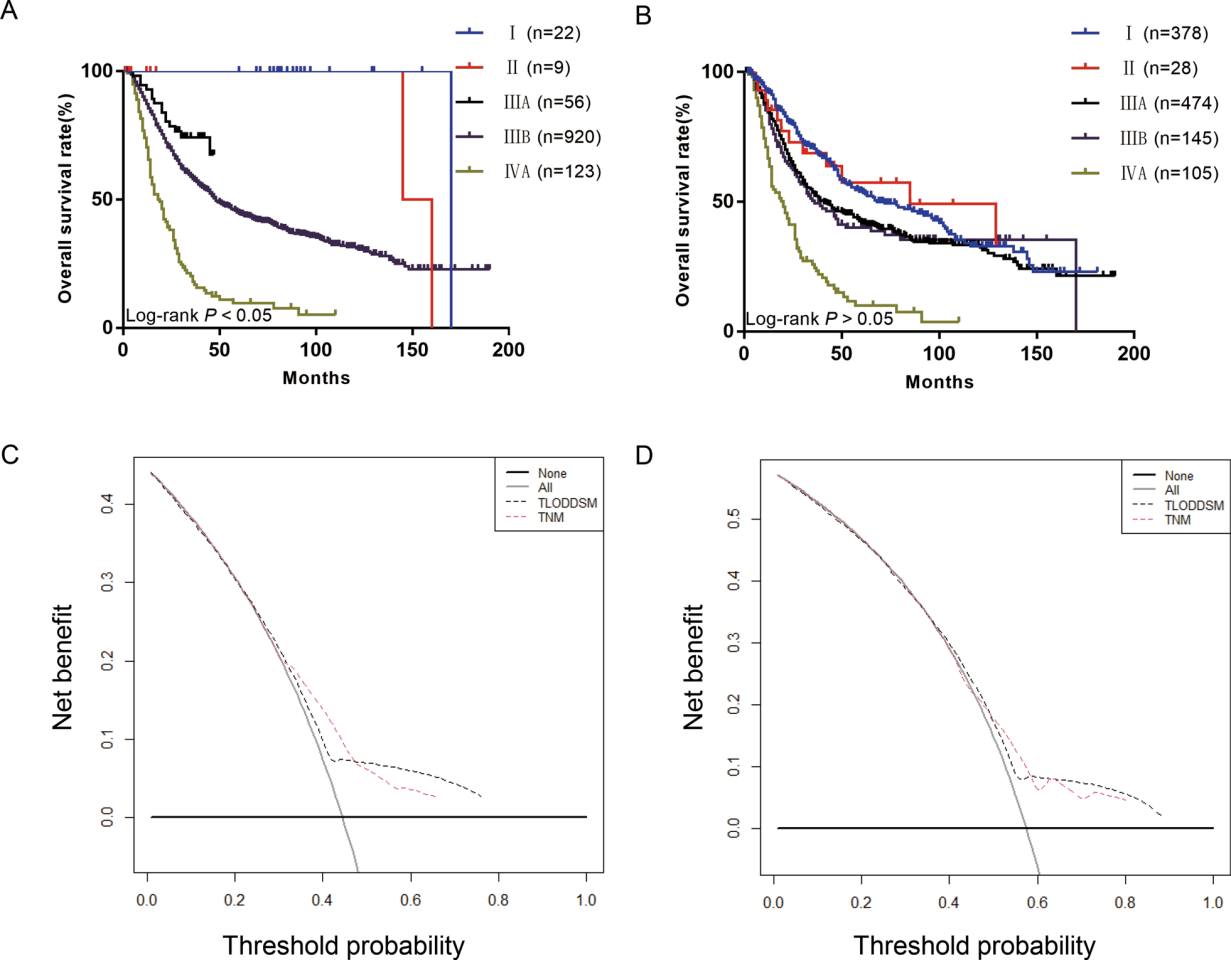
**A** The Kaplan–Meier curves of OS for TLODDSM staging system; **B** The Kaplan–Meier curves of OS for traditional TNM staging system; **C** TLODDSM stage were compared to the TNM stage in terms of 3-year OS in our DCA; **D** TLODDSM stage were compared to the TNM stage in terms of 5-year OS in our DCA

As the number of ELNs increased, the patient’s prognosis improved (Fig. 2A); however, as the LODDS increased, the patient’s prognosis worsened (Fig. 2B). The range of LODDS was − 2.1–1.8, and we divided LODDS into 17 groups at intervals of 0.2. The HR of − 2.1 ≤ LODDS ≤ − 2.0 was defined as 1, and the HR values for each stage were calculated and sorted from low to high, with the highest as 1.0 < LODDS ≤ 1.8 (HR = 7.875; 95% CI 1.672–37.095; P = 0.009). Survival analysis was performed on 17 consecutive stages, and chi square values were calculated. Four high chi square values were identified as cutoff points (4.207, 1.265, 0.392, 0.265), and patients were divided into five stages as follows (Table 2). The 5-year survival rates for LODDS1–5 were 71.87, 57.80, 35.14, 24.14, and 16.46%, respectively, with statistically significant differences, indicating that our staging system has a high discriminative ability (Fig. 2C). According to the eighth edition of staging, we used the LODDS staging system instead of the traditional staging system to divide patients into 35 groups. The HR of T0LODDS1M0 was defined as 1, and the HR values for each stage were calculated and sorted from low to high, with the highest as T4bLODDS5M0 (HR = 398.007; 95% CI 24.479–6471.206; P < 0.001; Table 3). Survival analysis was performed on 35 consecutive stages (Fig. 3A), and chi square values were calculated. Four high chi square values were identified as cutoff points (1.958, 1.007, 1.332, 0.636), and patients were divided into five stages as follows (Fig. 3B): stage I (T0LODDS1), stage II (TisLODDS1), stage IIIA (T1LODDS1, T2LODDS1), IIIB (T3LODDS1, T0LODDS2, TisLODDS2, T1LODDS2, T2LODDS2, T0LODDS3, TisLODDS3, T0LODDS4, T1LODDS3, T2LODDS3, T3LODDS2, T4aLODDS1, TisLODDS4, T0LODDS5, T3LODDS, TisLODDS5, T1LODDS4), and stage IVA (T4aLODDS2, T1LODDS5, T4bLODDS1, T2LODDS4, T4aLODDS3, T3LODDS4, T4bLODDS2, T2LODDS5, T3LODDS5, T4bLODDS3, T4aLODDS4, T4aLODDS5, T4bLODDS4, T4bLODDS5). The HR of stage I was 1. The 5-year survival rates of stage II (HR = 11.118; 95% CI 1.005–123.042; P < 0.05), stage IIIA (HR = 13.058; 95% CI 1.722–99.014; P = 0.013), stage IIIB (HR = 22.675; 95% CI 3.186–161.352; P = 0.002), and stage IVA (HR = 63.741; 95% CI 8.872–457.934; P < 0.001) were 100.00, 100.00, 67.53, 44.58, and 9.60%, respectively, with statistically significant differences (P < 0.05; Fig. 4A). However, the 5-year survival rates of stage I to stage IVA in the traditional TNM staging system were 53.98, 57.42, 41.89, 39.99, and 9.98%, respectively, with no statistically significant differences (P > 0.05; Table 4).

**Table 2:** The COX analyses of LODDS groups in the training set

Variable	Univariate COX regression		Multivariate COX regression	
	HR (95% CI)	P-value	χ^2^	P-value
LODDS1				
− 2.1 ≤ LODDS ≤ − 2.0	1			
− 2.0 < LODDS ≤ − 1.8	0.688 (0.149–3.184)	0.632	0.327	0.567
LODDS2				
− 1.8 < LODDS ≤ − 1.6	1.306(0.319–5.349)	0.710	4.207	0.040
− 1.6 < LODDS ≤ − 1.4	1.450 (0.357–5.886)	0.603	0.212	0.645
LODDS3				
− 1.4 < LODDS ≤ − 1.2	1.758 (0.426–7.259)	0.436	1.265	0.261
− 1.2 < LODDS ≤ − 1.0	2.081 (0.511–8.471)	0.306	0.530	0.467
− 1.0 < LODDS ≤ − 0.8	2.156 (0.527–8.826)	0.285	0.026	0.872
− 0.8 < LODDS ≤ − 0.6	2.609 (0.478–14.248)	0.268	0.143	0.706
− 0.6 < LODDS ≤ − 0.4	3.111 (0.758–12.766)	0.115	0.036	0.849
− 0.4 < LODDS ≤ − 0.2	3.153 (0.771–12.904)	0.110	0.002	0.961
LODDS4				
− 0.2 < LODDS ≤ 0	3.575 (0.862–14.839)	0.079	0.392	0.531
0 < LODDS ≤ 0.2	3.744 (0.872–16.078)	0.076	0.003	0.957
LODDS5				
0.2 < LODDS ≤ 0.4	4.299 (1.035–17.859)	0.045	0.265	0.606
0.4 < LODDS ≤ 0.6	5.108 (1.084–24.063)	0.039	0.325	0.569
0.6 < LODDS ≤ 0.8	6.239 (1.426–27.292)	0.015	0.069	0.793
0.8 < LODDS ≤ 1.0	6.443 (1.248–33.251)	0.026	0.025	0.874
1.0 < LODDS ≤ 1.8	7.875 (1.672–37.095)	0.009	0.149	0.700

*LODDS* logarithmic odds of positive lymph nodes

**Table 3:** The COX analyses of TLODDSM groups in the training set

Variable	Univariate COX regression		Multivariate COX regression	
	HR (95% CI)	P-value	χ^2^	P-value
Stage I				
T0 LODDS1	1			
Stage II				
Tis LODDS1	11.727 (1.062–129.527)	0.045	1.958	0.162
Stage IIIA				
T1 LODDS1	12.256 (1.109–135.398)	0.041	1.007	0.316
T2 LODDS1	12.282 (0.767–196.571)	0.076	0.003	0.959
Stage IIIB				
T3 LODDS1	12.686 (1.560–103.133)	0.017	1.332	0.248
T0 LODDS2	13.565 (1.772–103.876)	0.041	< 0.001	0.985
Tis LODDS2	13.979 (0.873–223.915)	0.062	0.001	0.971
T1 LODDS2	15.598 (2.165–112.380)	0.006	< 0.001	0.992
T2 LODDS2	17.559 (2.421–127.361)	0.005	0.396	0.529
T0 LODDS3	17.906 (2.424–132.250)	0.005	0.142	0.707
Tis LODDS3	22.912 (2.074–253.090)	0.011	0.359	0.549
T0 LODDS4	24.821 (3.458–178.146)	0.001	0.095	0.758
T1 LODDS3	26.878 (3.758–192.217)	0.001	0.320	0.571
T2 LODDS3	29.086 (3.874–218.353)	0.001	0.122	0.727
T3 LODDS2	30.534 (4.181–223.004)	0.001	0.567	0.451
T4a LODDS1	31.280 (1.952–501.263)	0.015	0.097	0.755
Tis LODDS4	34.541 (4.662–255.902)	0.001	0.084	0.773
T0 LODDS5	36.048 (4.204–309.097)	0.001	0.004	0.951
T3 LODDS3	36.193 (2.259–579.963)	0.011	0.014	0.905
Tis LODDS5	38.806 (4.668–322.583)	0.001	0.039	0.843
T1 LODDS4	41.003 (5.327–315.602)	< 0.001	0.044	0.833
Stage IVA				
T4a LODDS2	53.885 (7.223–402.008)	< 0.001	0.636	0.425
T1 LODDS5	55.117 (7.297–416.343)	< 0.001	< 0.001	0.984
T4b LODDS1	56.924 (5.911–548.169)	< 0.001	0.036	0.849
T2 LODDS4	59.707 (5.403–659.827)	0.001	0.004	0.951
T4a LODDS3	65.633 (8.930–482.379)	< 0.001	0.005	0.946
T3 LODDS4	68.075 (6.153–753.213)	0.001	0.012	0.914
T4b LODDS2	72.798 (8.487–624.428)	< 0.001	0.005	0.942
T2 LODDS5	90.649 (10.558–778.272)	< 0.001	0.098	0.754
T3 LODDS5	91.169 (8.249–1007.603)	< 0.001	0.085	0.771
T4b LODDS3	105.754 (9.561–1169.778)	< 0.001	0.154	0.695
T4a LODDS4	108.419 (12.078–973.259)	< 0.001	0.141	0.707
T4a LODDS5	131.544 (11.871–1457.603)	< 0.001	0.052	0.819
T4b LODDS4	137.238 (8.547–2203.649)	0.001	0.500	0.480
T4b LODDS5	398.007 (24.479–6471.206)	< 0.001	0.500	0.480

*LODDS* logarithmic odds of positive lymph nodes

**Table 4:** The COX analyses of different stage groups in the training set

Variable	AJCC 8th TNM		Current TLODDSM stage	
	HR (95% CI)	P-value	HR (95% CI)	P-value
Stage I	1		1	
Stage II	0.977 (0.544–1.758)	0.939	11.118 (1.005–123.042)	0.050
Stage IIIA	1.391 (1.143–1.693)	0.001	13.058 (1.722–99.014)	0.013
Stage IIIB	1.495 (1.138–1.964)	0.004	22.675 (3.186–161.352)	0.002
Stage IVA	3.492 (2.666–4.572)	< 0.001	63.741 (8.872–457.934)	< 0.001

*LODDS* logarithmic odds of positive lymph nodes

### Comparison of prognostic effectiveness between the two staging systems

The AIC (7356.862) and BIC (7361.242) of the TLODDSM staging system were lower than the AIC (7385.357) and BIC (7389.737) of the traditional TNM staging system, and the likelihood of the TLODDSM staging system was higher than that of the traditional TNM staging system (chi square test, 113.091 versus 95.953, Table 5). The DCA showed that the TLODDSM staging system had a higher net benefit compared to the traditional TNM staging system (Fig. 4C, D). Taken collectively, these results indicate that the TLODDSM staging system is superior to the AJCC staging system.

**Table 5:** Performance of the different staging systems for predicting prognosis

Stage	Likelihood ratio χ^2^	AIC	BIC	P-value
TNM (Training)	95.953	7385.357	7389.737	< 0.001
TLODDSM (Training)	113.091	7356.862	7361.242	< 0.001
TNM (External)	229.595	5285.257	5301.54	< 0.001
TLODDSM (External)	275.798	5229.22	5245.503	< 0.001

*LODDS* logarithmic odds of positive lymph nodes, *AIC* Akaike information, *BIC* Bayesian information criterion

### Selection of a population that can benefit from adjuvant chemotherapy

**Fig. 5:**
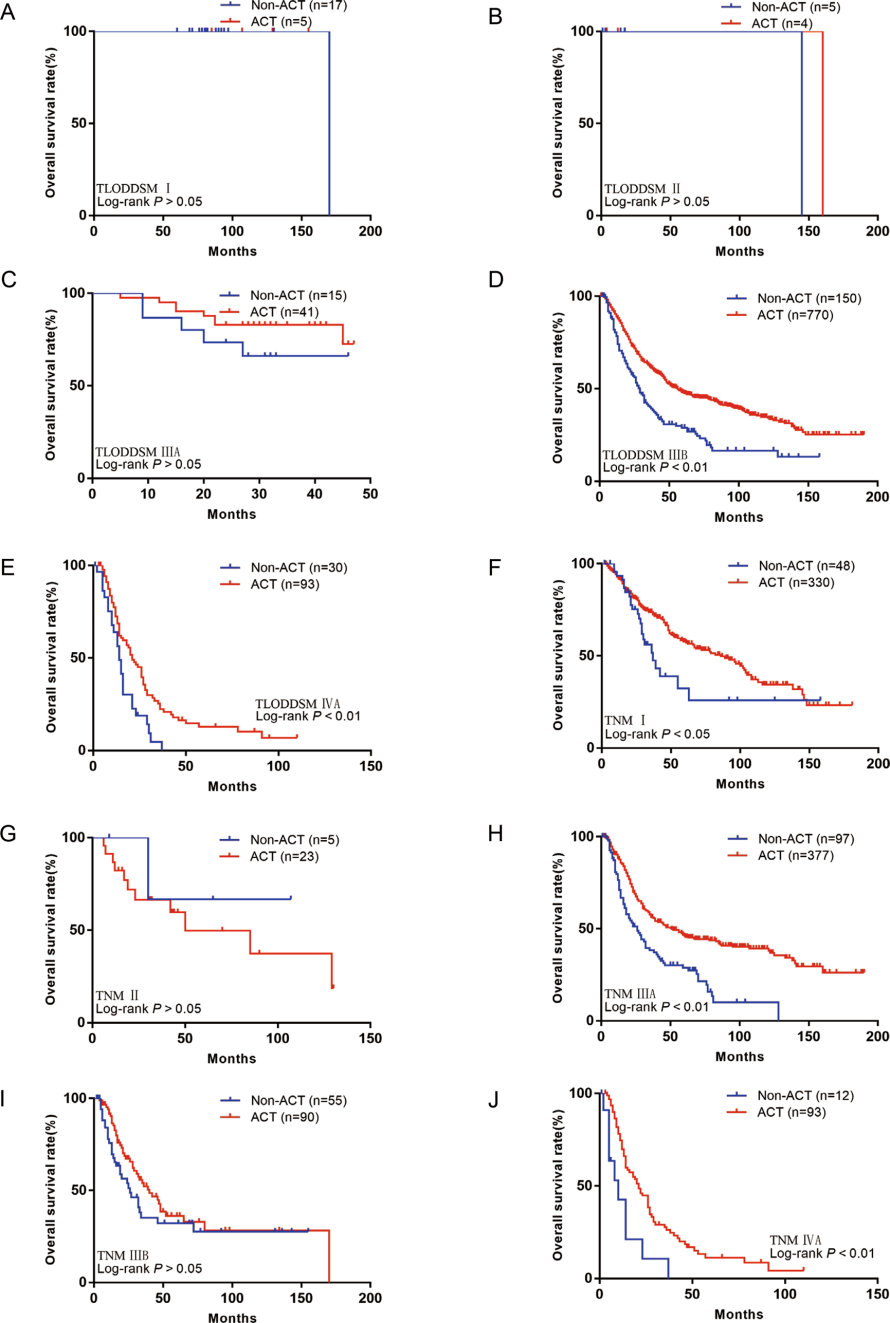
**A** OS for patients with or without ACT in TLODDSM stage I group in our training set; **B** OS for patients with or without ACT in TLODDSM stage II group in our training set; **C** OS for patients with or without ACT in TLODDSM stage IIIA group in our training set; **D** OS for patients with or without ACT in TLODDSM stage IIIB group in our training set; **E** OS for patients with or without ACT in TLODDSM stage IVA group in our training set; **F** OS for patients with or without ACT in TNM stage I group in our training set; **G** OS for patients with or without ACT in TNM stage II group in our training set; **H** OS for patients with or without ACT in TNM stage IIIA group in our training set; **I** OS for patients with or without ACT in TNM stage IIIB group in our training set; **J** OS for patients with or without ACT in TNM stage IVA group in our training set

Patients in stage I of the TLODDSM staging system did not benefit from ACT (P > 0.05; Fig. 5A). Furthermore, stage-II patients did not benefit from ACT (HR = 0.33; 95% CI 0.01–8.18; P > 0.05; Fig. 5B), stage-IIIA patients did not benefit from ACT (HR = 0.54; 95% CI 0.16–1.90; P > 0.05; Fig. 5C), stage-IIIB patients did benefit from ACT (HR = 0.55; 95% CI 0.42–0.71; P < 0.01; Fig. 5D), and stage-IVA patients did benefit from ACT (HR = 0.53; 95% CI 0.31–0.90; P < 0.01; Fig. 5E).

Patients in stage I of the traditional TNM staging system did benefit from ACT (HR = 0.61; 95% CI 0.36–1.02; P < 0.05; Fig. 5F). Furthermore, stage-II patients did not benefit from ACT (HR = 2.07; 95% CI 0.44–9.74; P > 0.05; Fig. 5G), stage-IIIA patients did benefit from ACT (HR = 0.51; 95% CI 0.37–0.70; P < 0.01; Fig. 5H), stage-IIIB patients did not benefit from ACT (HR = 0.75; 95% CI 0.47–1.21; P > 0.05; Fig. 5I), and stage-IVA patients did benefit from ACT (HR = 0.39; 95% CI 0.15–1.04; P < 0.01; Fig. 5J).

### T LODDS M staging system in the external validation group

**Fig. 6:**
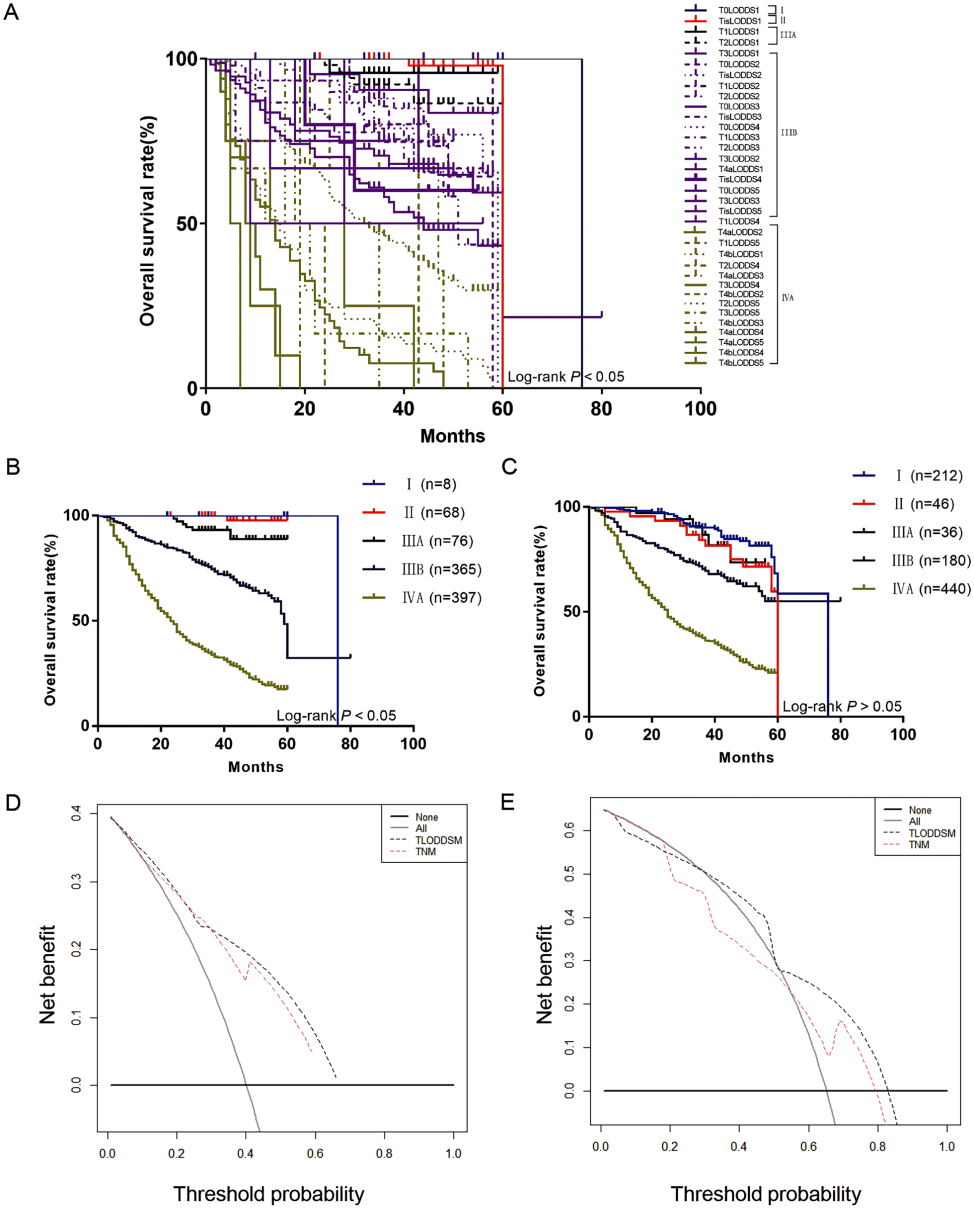
**A** The Kaplan–Meier curves of OS for patients in our new LODDS staging system in our external set; **B** The Kaplan–Meier curves of OS for TLODDSM staging system in our external set; **C** The Kaplan–Meier curves of OS for traditional TNM staging system in our external set; **D** TLODDSM stage were compared to the TNM stage in terms of 3-year OS in our DCA in our external set; **E** TLODDSM stage were compared to the TNM stage in terms of 5-year OS in our DCA in our external set

After considering the inclusion criteria, 914 patients with resectable GEC who received neoadjuvant chemoradiation in our hospital were included. Specifically, 297 (32.5%) patients were 65 years of age or older, 268 (29.3%) patients were female, 413 (45.2%) patients had poorly differentiated tumors, 377 (41.2%) patients had tumors with dimeters greater than 5 cm, and 472 (51.6%) patients had T4 tumors. The median number of ELNs was 18, and the median number of PLNs was 2 (Table 1). The median survival period was 35 months (range, 0–80), and the number of deaths was 433 (47.4%). According to the above criteria, the patients were further divided into 35 groups and classified into five stages based on the TLODDSM staging system (Fig. 6A). Stages I, II, IIIA, IIIB, and IVA had 5-year survival rates of 100.00, 97.78, 88.91, 32.33, and 17.44%, respectively, with statistically significant differences (P < 0.05, Fig. 6B). The 5-year survival rates of stage I to IVA in the traditional TNM staging system were 55.70, 0, 73.54, 55.04, and 20.99%, respectively, with no statistically significant differences (P > 0.05; Fig. 6C).

The AIC (5229.220) and BIC (5245.503) of the TLODDSM staging system were lower than the AIC (5285.257) and BIC (5301.54) of the traditional TNM staging system, and the likelihood of the new staging system was higher than that of the old staging system (chi-square test, 275.798 versus 229.595, Table 5). The DCA showed that the TLODDSM staging system had a higher net benefit compared to the traditional TNM staging (Fig. 6D, E). The externally validated data from our center supported this conclusion.

**Fig. 7:**
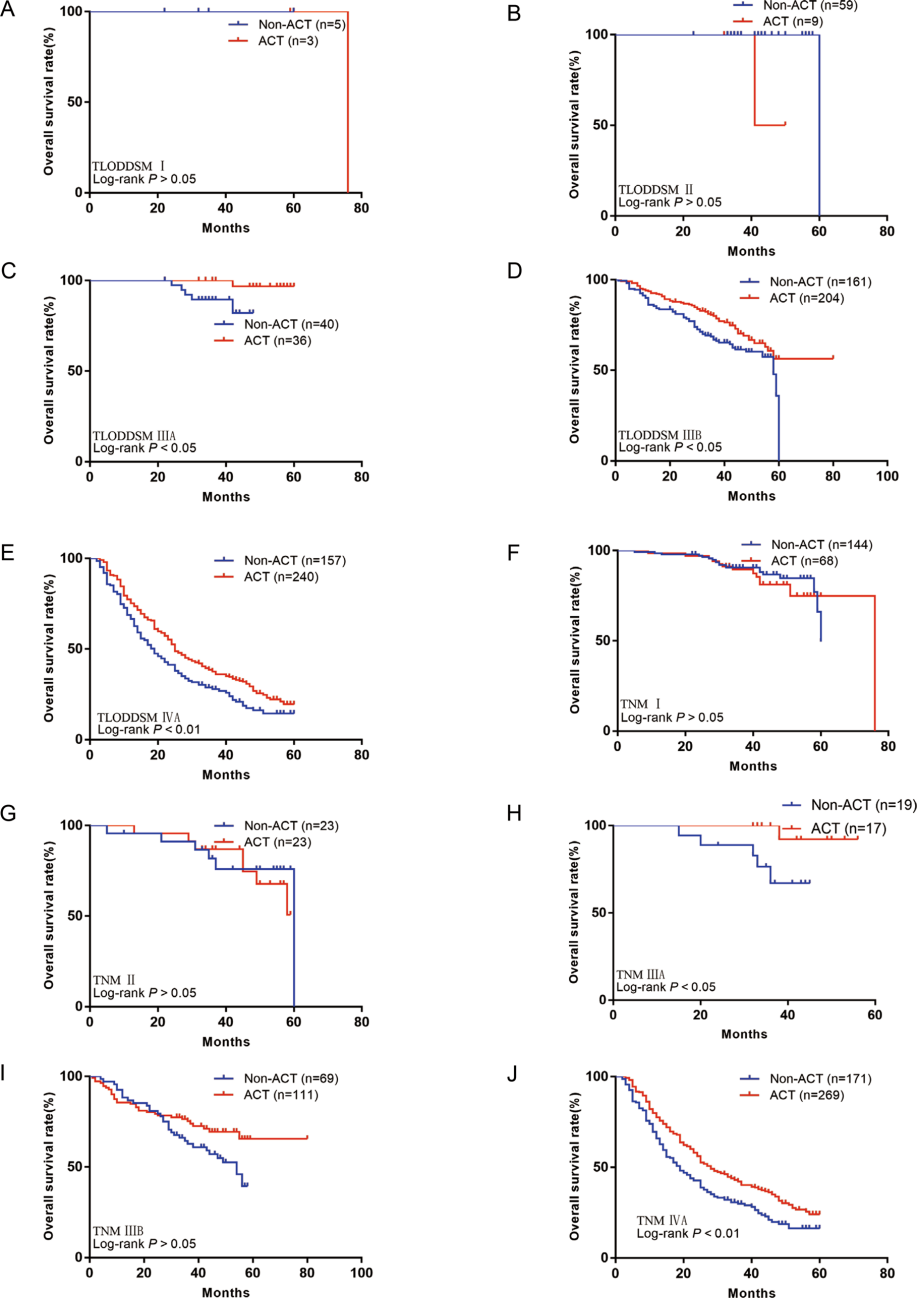
**A** OS for patients with or without ACT in TLODDSM stage I group in our external set; **B** OS for patients with or without ACT in TLODDSM stage II group in our external set; **C** OS for patients with or without ACT in TLODDSM stage IIIA group in our external set; **D** OS for patients with or without ACT in TLODDSM stage IIIB group in our external set; **E** OS for patients with or without ACT in TLODDSM stage IVA group in our external set; **F** OS for patients with or without ACT in TNM stage I group in our external set; **G** OS for patients with or without ACT in TNM stage II group in our external set; **H** OS for patients with or without ACT in TNM stage IIIA group in our external set; **I** OS for patients with or without ACT in TNM stage IIIB group in our external set; **J** OS for patients with or without ACT in TNM stage IVA group in our external set

The TLODDSM staging system showed that stage-I patients did not benefit from ACT (P > 0.05; Fig. 7A), stage-II patients did not benefit from ACT (P > 0.05; Fig. 7B), stage-IIIA patients did benefit from ACT (HR = 0.15; 95% CI 0.03–0.77; P < 0.05; Fig. 7C), stage-IIIB patients did benefit from ACT (HR = 0.67; 95% CI 0.47–0.96; P < 0.05; Fig. 7D), and stage-IVA patients did benefit from ACT (HR = 0.73; 95% CI 0.57–0.93; P < 0.01; Fig. 7E).

The traditional TNM staging system showed that stage-I patients did not benefit from ACT (HR = 1.16; 95% CI 0.57–2.37; P > 0.05; Fig. 7F), stage-II patients did not benefit from ACT (HR = 1.20; 95% CI 0.40–3.55; P > 0.05; Fig. 7G), stage-IIIA patients did benefit from ACT (HR = 0.15; 95% CI 0.03–0.74; P < 0.05; Fig. 7H), stage-IIIB patients did not benefit from ACT (HR = 0.62; 95% CI 0.38–1.03; P > 0.05; Fig. 7I), and stage-IVA patients did benefit from ACT (HR = 0.68; 95% CI 0.54–0.87; P < 0.01; Fig. 7J).

## Discussion

The eighth edition of the TNM Staging System for Esophageal Cancer and GEC distinguishes clinical staging (cTNM), pathological staging (pTNM), and neoadjuvant treatment staging (ypTNM), and no longer applies one staging model. Unlike cTNM and pTNM staging, ypTNM staging for the two pathological types, adenocarcinoma and squamous cell carcinoma, is identical [11–13]. For the staging of GEC, the seventh edition continues to be used, which emphasizes PLN as the node staging standard, without reflecting the impact of the lymph node station number on staging [14, 15]. Although many studies support the correlation between the lymph node stage number and GEC prognosis, some lymph nodes may fuse due to the invasiveness of the tumor, or some PLNs may rupture during surgical dissection, resulting in inaccurate node staging based on the number. Furthermore, neoadjuvant chemoradiation may result in a decreased number of ELNs [16–18]. Therefore, a new node staging system is needed to more accurately evaluate the prognosis of patients. New staging systems for lymph nodes, such as the lymph node ratio (LNR), LODDS, negative examined lymph node (NLN) number, and lymph node micro-metastases, can better predict the prognosis of patients [19–21]. According to Yang et al., in the context of a new TNM staging system, LODDS has better predictive value than node number, LNR, and NLN number in patients with PLNs [11].

LODDS is defined as the logarithm of the probability ratio of positive or negative lymph nodes when one lymph node is detected, which effectively balances the number of ELNs and the number of PLNs [22]. Spolverato et al. reported that the LODDS system (C index: 0.636; AIC: 4304.0) was more sensitive than the node score system (C index: 0.632; AIC: 4308.4) and the LNR system (C index: 0.631; AIC: 4225.8) in 804 patients who underwent radical gastrectomy for gastric cancer [23]. Among Siewert type II gastroesophageal adenocarcinoma patients who underwent surgery after neoadjuvant radiotherapy, the LNR was more sensitive than the PLN number, and LODDS was an independent prognostic factor (HR = 1.16; 95% CI 1.08–1.25; P < 0.01) [24]. Zhou et al. demonstrated that LODDS (C index: 0.675; AIC: 6243.740) and LNR (C index: 0.686; AIC: 6261.027) staging systems were superior to the traditional node staging system (C index: 0.658; AIC: 6355.077) in predicting the prognosis of 735 patients with gastroesophageal adenocarcinoma who completed surgery, and externally validated data also confirmed this conclusion [25]. To reduce the stage migration caused by a decreased ELN number [26–28], we replaced the traditional TNM staging system with the LODDS staging system (LODDS1–5). The overall survival of patients was significantly improved with the LODDS1–5 staging system, and stages I, II, IIIA, IIIB, and IVA showed statistical differences in the 5-year survival rate, whereas the traditional TNM staging system showed no statistical differences, indicating that the new staging system can better predict the prognosis of patients. Furthermore, we compared the AIC, BIC, and DC of the two staging systems and observed that the heterogeneity in the TLODDSM staging system was relatively low, making it the best staging system for evaluating patient prognosis.

Different stage combinations can lead to completely different prognoses, and it is unclear whether patients with GEC who receive neoadjuvant chemoradiation can benefit from ACT. Rahman et al. reported that patients with GEC who received neoadjuvant chemotherapy benefited from ACT (HR = 0.84; 95% CI 0.77–0.94; P = 0.001) [29]. Mokdad et al. [30] studied 10 086 patients with gastroesophageal adenocarcinoma who received neoadjuvant chemotherapy and radiotherapy in 2006 and 2013 and reported that ACT improved the OS (HR = 0.79; 95% CI 0.72–0.88; P < 0.001) and median survival (40 versus 34 months). On the other hand, Sisic et al. observed no improvement in OS (P = 0.331) and relapse-free survival (P = 0.118) in patients with GEC receiving ACT [31], suggesting not all patients require ACT. Our study shows that stage-I, -II, and -IIIA patients did not benefit from ACT, whereas stage-IIIB and -IVA patients did benefit from treatment. In terms of the traditional TNM staging system, stage- I, -IIIA, and -IVA patients did benefit from ACT, whereas stage-II and -IIIB patients did not benefit from treatment. Our study also shows that the poorer the staging, the more likely the patient is to benefit from ACT. However, traditional TNM stages do not imply that patients with poorer prognosis are more likely to benefit from ACT, indicating that our staging system can guide the postoperative monitoring, follow-up, and prognosis of patients and avoid the consequences of over-treatment and under-treatment.

This study has several limitations. First, the inconsistency of neoadjuvant radiation dosage and chemotherapy regimen can lead to certain biases. Second, different centers, different pathologists, and the lack of unified standards for lymph node detection can also lead to bias. Third, this study was a retrospective study. The optimal cut-off value for the new staging system is still difficult to reach consensus, and when referring to LODDS for staging, the calculation is not fast and convenient. Except for patients with a higher risk of lymph node staging migration, it may not significantly improve the predictive prognosis. Presently, there is no standard protocol for the staging and treatment of GEC after receiving neoadjuvant chemoradiation. However, we established the LODDS staging system to re-stage patients, which can guide treatment and predict prognosis.

## Conclusion

The TLODDSM staging system is superior in staging and predicting the prognosis of patients with GEC after receiving neoadjuvant chemoradiation (radiation first). We recommend that stage-IIIB and -IVA patients should receive ACT.

## Data Availability

Data will be made available on request.

## Abbreviations

GEC: Gastroesophageal cancer
ELNs: Examined lymph nodes
OS: Overall survival
PFS: Progression-free survival
ACT: Adjuvant chemotherapy
LODDS: Logarithmic odds of positive lymph nodes
PLN: Positive lymph nodes
AIC: Akaike information criterion
BIC: Bayesian information criterion
DCA: Decision curve analysis
NLN: Negative examined lymph node
